# Percutaneous Radiofrequency Ablation Combined With Balloon Kyphoplasty for Painful Vertebral Metastasis and Acute Pathologic Compression Fracture: A Case Report

**DOI:** 10.7759/cureus.102717

**Published:** 2026-01-31

**Authors:** Renato Abu Hana, Ruben G Ortiz Cordero, Oswaldo A Guevara Tirado, Grit A Adler, Vinicius Adami Vayego Fornazari

**Affiliations:** 1 Division of Vascular and Interventional Radiology, Department of Radiology, College of Medicine, University of Florida, Jacksonville, USA

**Keywords:** kyphoplasty, palliative care, pathologic compression fracture, radiofrequency ablation, vertebral cement augmentation, vertebral metastases

## Abstract

Painful vertebral bone metastases may result in pathologic compression fractures, severe mechanical back pain, and functional impairment. Conventional external beam radiotherapy is commonly used for palliation but is limited by delayed analgesic effects and the inability to address spinal instability. Minimally invasive image-guided techniques, such as percutaneous radiofrequency ablation (RFA) combined with vertebral cement augmentation (VCA), may provide rapid pain relief while restoring mechanical stability.

We report the case of a 66-year-old man with advanced lung cancer who presented with severe opioid-refractory low back pain. Magnetic resonance imaging demonstrated an acute L4 pathologic compression fracture with approximately 40% vertebral height loss and no evidence of spinal cord compression. The patient underwent a same-session image-guided percutaneous vertebral biopsy followed by RFA and balloon kyphoplasty. Pre-procedural pain severity was assessed using the Visual Analog Scale (VAS), with a score of 10/10. Within 24 hours after the intervention, the patient reported complete pain relief (VAS 0/10) and was able to ambulate independently, without procedural complications.

This case highlights same-session percutaneous RFA combined with VCA as a minimally invasive strategy capable of providing rapid pain relief and immediate spinal stabilization in selected patients with painful vertebral metastases and pathologic compression fractures without spinal cord compression.

## Introduction

Vertebral bony metastases (VBM) are the most common form of skeletal metastasis, affecting 20% of all patients with cancer and up to 40% of patients with metastatic disease [1-3]. VBM more commonly occur in the thoracic spine, followed by the lumbosacral and cervical spine [2]. Patients with VBM can present with debilitating back pain, limited mobility, vertebral fracture or collapse, weakness, paresthesia, and metastatic spinal cord compression [2,4,5]. Given their limited life expectancy, often less than one year, the management of patients with VBM is mostly palliative, primarily seeking to provide pain relief, spinal stability, and an improved quality of life [1,2]. Treatment options for patients with symptomatic VBM include bisphosphonates, steroids, chemotherapy, radiotherapy, and surgical management [2,6]. Currently, conventional external beam radiotherapy (cEBRT) is the standard of care for palliation of painful bone metastases in the absence of vertebral instability or evidence of spinal cord compression [3,4]. However, cEBRT has several limitations, including limited and delayed pain relief, recurrence of symptoms, and potential exclusion of patients from receiving certain systemic chemotherapies [3,4]. On the other hand, surgical management is highly invasive and carries increased risk for complications [7].

Achieving rapid and durable pain control in patients with opioid-resistant vertebral metastases and acute pathologic fractures remains challenging, as radiotherapy often provides only partial relief with delayed onset [3,4]. Radiofrequency ablation (RFA) of vertebral tumors and vertebral cement augmentation (VCA), including kyphoplasty, have emerged as minimally invasive alternatives that provide effective pain relief while preserving vertebral stability [5]. RFA is primarily performed for the targeted thermal debulking of tumor tissue rather than for the direct denervation of basivertebral or periosteal nerves [3-5]. During the procedure, radiofrequency electrodes are positioned within the tumor-bearing portion of the vertebral body to create a controlled zone of coagulative necrosis [3-5]. This targeted thermal injury results in the destruction of tumor-associated nociceptive fibers, reduction of tumor burden and intravertebral pressure, and decreased local release of inflammatory and algogenic mediators [3,4,7]. The ablation zone may also disrupt intraosseous and periosteal sensory nerve fibers [3,4,7]. When combined with cement augmentation, additional pain relief is achieved through mechanical stabilization and elimination of painful micromotion, with a potential supplementary neurolytic effect from the exothermic polymerization of polymethylmethacrylate [5,6].

Kyphoplasty further contributes to pain relief in patients with VBM-related vertebral fractures by restoring vertebral height and stability through balloon cavity creation followed by cement injection [5,7]. The combination of RFA and VCA has been shown to be a safe and effective strategy for rapid pain relief (often within 24 hours) with low complication rates [3,5,7,8]. Performing RFA and VCA during the same procedural session eliminates the need for multiple anesthetic events, reduces overall procedure time, and allows immediate vertebral reinforcement following tumor debulking, thereby decreasing the risk of post-ablation vertebral fracture [3,4]. Given the limited prospective data on combined RFA and vertebral augmentation, detailed case reports of same-session treatment for acute pathologic compression fractures remain valuable in demonstrating technical feasibility and clinical efficacy [4,5]. The purpose of this case report is to highlight the role of same-session image-guided percutaneous RFA combined with VCA as a minimally invasive strategy for rapid pain relief and mechanical stabilization in patients with painful vertebral metastases and pathologic compression fractures without spinal cord compression.

## Case presentation

A 66-year-old man with a past medical history of lung cancer and laryngeal neoplasm presented to the emergency department with severe intractable low back pain. The patient reported the pain starting two weeks prior to admission and described it as 10/10 in pain severity on the Visual Analog Scale (VAS), non-radiating, and worsening with movement. He denied symptoms of weakness, incontinence, or paresthesia. He had been using a fentanyl patch and morphine to manage his pain; however, symptoms persisted and were now limiting his mobility. Physical examination was remarkable for lower back pain without myalgias or arthralgias. His complete blood count was remarkable for mild thrombocytopenia (Table 1). The comprehensive metabolic panel was remarkable for elevated blood urea nitrogen (Table 2).

**Table 1:** Complete blood count

Test	Result	Range	Unit
Hemoglobin	13.6	14.0-18.0	g/dL
Hematocrit	39.6	40.0-54.0	%
White blood cells	11.67	4.5-11	thou/cumm
Platelet count	109	140-440	×10^3^/µL

**Table 2:** Comprehensive metabolic panel

Test	Result	Range	Unit
Sodium	135	135-145	mmol/L
Potassium	4.7	3.4-4.5	mmol/L
Chloride	101	98-107	mmol/L
Carbon dioxide	21	21-29	mmol/L
Glucose	123	71-99	mg/dL
Urea nitrogen	35.5	6.0-22.0	mg/dL
Creatinine	0.92	0.67-1.17	mg/dL
Albumin	3.7	3.8-4.9	g/dL
Total bilirubin	0.6	0.2-1.0	mg/dL
Alanine aminotransferase	17	10-42	IU/L
Aspartate aminotransferase	24	14-33	IU/L
Estimated glomerular filtration rate	90	≥60	mL/min/1.73 m^2^

**Figure 1:**
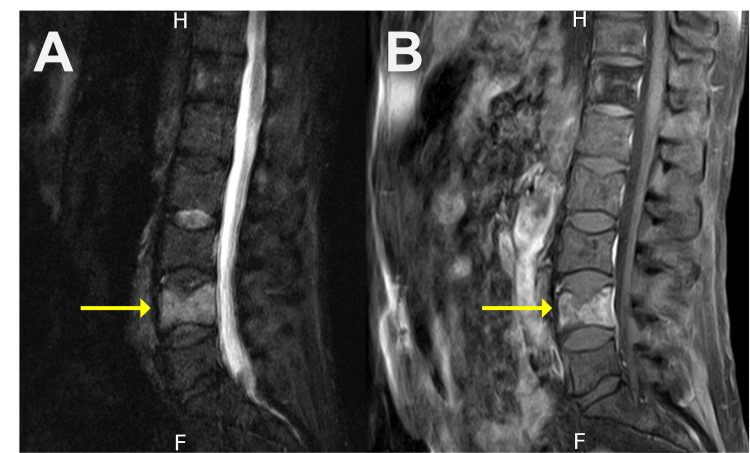
(A) Sagittal short tau inversion recovery and (B) sagittal T1-weighted fast spin echo with fat saturation and intravenous contrast magnetic resonance imaging showing an acute L4 vertebral body fracture with 40% height loss (yellow arrows)

Magnetic resonance imaging (MRI) revealed multiple metastatic lesions throughout the spine, as well as an acute pathologic compression fracture of L4 with 40% vertebral height loss (Figure 1A-1B). After evaluation, the Eastern Cooperative Oncology Group (ECOG) performance status was determined to be 3, and the Spinal Instability Neoplastic Score (SINS) was 8, consistent with potential instability. Interventional radiology was consulted for pain management, and after obtaining informed consent, we proceeded with an RFA, kyphoplasty, and biopsy of the L4 vertebral body with the Stryker OptaBlate Ablation System (Stryker Corporation, Kalamazoo, Michigan, United States). The procedure was done under moderate sedation and using the maximal sterile barrier technique. The L4 vertebral body was identified fluoroscopically. Then, after administering 2% lidocaine, two 10-gauge needles were introduced into the L4 vertebrae via a bilateral transpedicular approach and placed in the posterior third of the vertebral body. Next, a core biopsy of the vertebral body was performed from each of the access cannulas.

**Figure 2:**
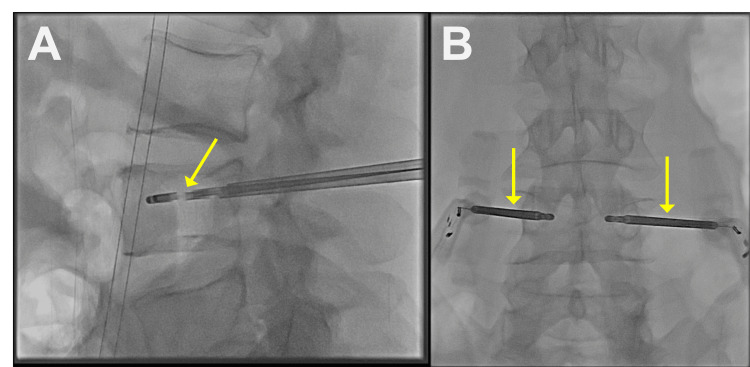
Computed tomography fluoroscopic imaging showing radiofrequency ablation probes inserted into the L4 vertebral body (yellow arrows)

After the biopsy was collected, a hand drill was advanced to the anterior aspect of the vertebral body and used to create a cavity for the RFA probes (Figure 2A-2B). Next, the 20 mm RFA probes were inserted into the cannulas and advanced to the anterior aspects of the vertebral bodies. RFA of the L4 vertebral body was performed at 95°C for 10 minutes with continuous micro-infusion (saline) technology which delivers a regulated flow of sterile saline to the ablation zone (6-10 mL/H).

**Figure 3:**
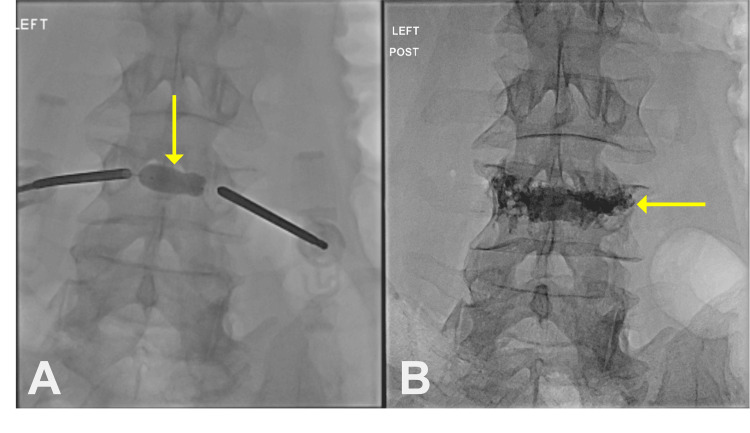
Computed tomography fluoroscopic imaging showing (A) the inflated balloon creating a cement cavity in the L4 vertebral body (yellow arrow) and (B) the L4 vertebral body filled with cement (yellow arrow)

Once the RFA was completed, the probes were removed, and a unipedicular curved balloon kyphoplasty system (Stryker OmniCurve Vertebral Balloon System, Stryker Corporation, Kalamazoo, Michigan, United States) was inserted through left pedicular access. The balloon was selected based on target cavity size (e.g., ~15-30 mm nominal tamp lengths) and inflated under fluoroscopic visualization to achieve adequate trabecular compaction and space creation while monitoring balloon integrity (Figure 3A). The balloon was then removed, and the L4 vertebral body was slowly filled with cement under direct fluoroscopy visualization (Figure 3B). The patient tolerated the procedure without immediate complications.

The following day, the patient stated complete resolution of his lower back pain, reporting a VAS score of 0/10 for pain severity, and was able to ambulate independently with an improved ECOG performance status of 1. However, due to the patient's advanced cancer staging, he was discharged to hospice care to continue comfort-focused care.

## Discussion

The management of painful VBM is clinically challenging and often requires multimodal therapy, including radiotherapy (e.g., cEBRT) or surgical intervention [1,5]. However, cEBRT has limited and delayed pain relief with frequent recurrence of symptoms, while surgical options have high complication rates [3,4,7]. Percutaneous RFA and VCA, such as kyphoplasty, are minimally invasive treatment options for patients suffering from painful VBM [3,6,7,9]. This case demonstrates a successful use of combined RFA and kyphoplasty for rapid and substantial pain relief in a patient presenting with severe, opioid-resistant, mechanical low back pain due to an acute L4 pathologic compression fracture without evidence of spinal cord compression. Current literature supports the use of combined RFA and VCA (e.g., kyphoplasty) for patients with vertebral metastases and pathologic compression fractures who have debilitating back pain in the absence of spinal cord compression [3,4]. Systematic reviews have shown that most patients achieve significant pain relief, with mean pain scores dropping significantly within 24-72 hours, while total complication rates remain low, ranging from 1% to 3% with major complications occurring in less than 0.5% of cases [5,9]. This combined approach addresses tumor-related pain and provides spinal stability and rapid pain relief with minimal complications [3,4,6]. The immediate analgesic effect results from the thermal destruction of periosteal nociceptors during RFA combined with instantaneous mechanical stabilization from cement augmentation [5].

Despite the effectiveness of RFA and VCA in managing VBM, it is important to discuss their limitations. For instance, using RFA alone can increase the risk of spinal instability and subsequent vertebral body fracture and collapse due to the formation of a cavity within the vertebral body after tumor debulking [4]. There are reports of non-augmented, RFA-treated vertebrae fracturing within 12 months after the procedure [3]. Thermal injury to healthy tissue can also occur in RFA but can be mitigated with adequate pre-procedural planning and thermal protection techniques [6,8]. Furthermore, standalone VCA can provide spinal stability for vertebral pathologic fractures [2,4,5,9]. However, if local tumor burden is not addressed with the use of RFA, symptom relief can be transient and suboptimal [2,4,5,9]. VCA also carries the risk of cement extravasation into the epidural space or neural foramina, leading to spinal cord compression [7]. This risk of cement extravasation can be mitigated by both microthrombosis of peritumoral venous vascularity and RFA-related cavity formation before cement injection [4,7]. Overall, combining RFA with VCA offers targeted tumor destruction and improved vertebral stability in the setting of pathologic vertebral fractures without requiring multiple interventions [3,4,6,8].

Providing both rapid pain relief and restoration of function is important for improving overall quality of life in patients with limited life expectancy who choose comfort-focused palliative care [2,7]. While this case shows rapid improvement, the brief follow-up period impedes the assessment of long-term durability and delayed complications. This case highlights the use of same-session image-guided percutaneous RFA and kyphoplasty to provide rapid pain relief and return of function in a patient with VBM and an acute pathologic compression fracture, without spinal cord compression. The results of this intervention also further support the available data demonstrating that combined RFA and VCA can achieve clinically significant pain improvement while providing vertebral stability [4,5]. However, the variability of vertebral tumor involvement and retrospective design of most available evidence limit generalizability, necessitating prospective multicenter trials for further validation.

## Conclusions

External beam radiotherapy for painful vertebral metastases is limited by delayed analgesia and lack of mechanical stabilization. This case demonstrates that percutaneous RFA combined with VCA is a minimally invasive option that can provide rapid pain relief and immediate spinal stability in selected patients with advanced malignancy.
